# Systematic Meta-Analysis of Computer-Aided Detection of Breast Cancer Using Hyperspectral Imaging

**DOI:** 10.3390/bioengineering11111060

**Published:** 2024-10-24

**Authors:** Joseph-Hang Leung, Riya Karmakar, Arvind Mukundan, Pacharasak Thongsit, Meei-Maan Chen, Wen-Yen Chang, Hsiang-Chen Wang

**Affiliations:** 1 Department of Radiology, Ditmanson Medical Foundation Chia-Yi Christian Hospital, Chiayi City 600566, Taiwan; 01289@cych.org.tw; 2 Department of Mechanical Engineering, National Chung Cheng University, 168, University Rd., Min Hsiung, Chiayi City 62102, Taiwan; karmakarriya345@gmail.com (R.K.); d09420003@ccu.edu.tw (A.M.); 3 Faculty of Mechanical Engineering, King Mongkut’s University of Technology North Bangkok, Pracharat 1 Road, Wongsawang, Bangsue, Bangkok 10800, Thailand; ppacharasak18@gmail.com; 4 Center for Innovative Research on Aging Society (CIRAS), National Chung Cheng University, 168, University Rd., Min Hsiung, Chiayi 62102, Taiwan; laicmm@ccu.edu.tw; 5 Department of General Surgery, Kaohsiung Armed Forces General Hospital, 2, Zhongzheng 1st.Rd., Lingya District, Kaohsiung City 80284, Taiwan; 6 Department of Medical Research, Dalin Tzu Chi Hospital, Buddhist Tzu Chi Medical Foundation, No. 2, Minsheng Road, Dalin, Chiayi 62247, Taiwan; 7 Hitspectra Intelligent Technology Co., Ltd., 4F., No. 2, Fuxing 4th Rd., Qianzhen Dist., Kaohsiung City 80661, Taiwan

**Keywords:** hyperspectral imaging, breast cancer, computer-aided detection, systematic meta-analysis, Deeks’ funnel chart, diagnostic test accuracy, forest charts

## Abstract

The most commonly occurring cancer in the world is breast cancer with more than 500,000 cases across the world. The detection mechanism for breast cancer is endoscopist-dependent and necessitates a skilled pathologist. However, in recent years many computer-aided diagnoses (CADs) have been used to diagnose and classify breast cancer using traditional RGB images that analyze the images only in three-color channels. Nevertheless, hyperspectral imaging (HSI) is a pioneering non-destructive testing (NDT) image-processing technique that can overcome the disadvantages of traditional image processing which analyzes the images in a wide-spectrum band. Eight studies were selected for systematic diagnostic test accuracy (DTA) analysis based on the results of the Quadas-2 tool. Each of these studies’ techniques is categorized according to the ethnicity of the data, the methodology employed, the wavelength that was used, the type of cancer diagnosed, and the year of publication. A Deeks’ funnel chart, forest charts, and accuracy plots were created. The results were statistically insignificant, and there was no heterogeneity among these studies. The methods and wavelength bands that were used with HSI technology to detect breast cancer provided high sensitivity, specificity, and accuracy. The meta-analysis of eight studies on breast cancer diagnosis using HSI methods reported average sensitivity, specificity, and accuracy of 78%, 89%, and 87%, respectively. The highest sensitivity and accuracy were achieved with SVM (95%), while CNN methods were the most commonly used but had lower sensitivity (65.43%). Statistical analyses, including meta-regression and Deeks’ funnel plots, showed no heterogeneity among the studies and highlighted the evolving performance of HSI techniques, especially after 2019.

## 1. Introduction

Globally, breast cancer is a common malignancy among women, accounting for over 570,000 deaths from the disease in 2015 [1]. The disparity in breast cancer rates of survival between advanced and developing nations is significant, with an approximate 5-year survival rate of 80% in affluent countries and less than 40% in underdeveloped ones [2]. For example, the American Cancer Society reports that there were 268,600 novel incidents of breast cancer in the year 2019, and the disease claimed the lives of almost 15% of these women [3]. Data from China show that the frequency rate also rises every year, and it is predicted that in the year 2050 about 3.2 million new cases per year worldwide will be recorded [4]. Moreover, not only is the number of patients increasing worldwide, but the age of affected patients is also tending to be younger [5]. Breast cancer is diagnosed based on a mammogram, abnormal lump in the breast, or variations in the nipple or skin [6]. It is found in the breast tissue, axillary lymph nodes, or more distant sites in the body [7]. The breast cancer risk factors can be classified into two categories: Inherent variables, such as sex, age, and race; The genetic makeup of the disease promotes a familial occurrence; and mammary gland benign proliferative lesions [8,9,10,11]. Another factor to consider is external variables, such as lifestyle choices and long-term medical interventions like hormonal contraception or replacement therapy. These factors can have a certain degree of influence on the development of neoplastic processes and can be adjusted to some extent [12,13,14,15,16]. The breast cancer diagnosis technique is operator-dependent and demands the skills of a veteran diagnostician. Nevertheless, many influences such as fatigue and a lack of attentiveness can be a source of misdetection, leading to low survival rates. To counteract this, CAD techniques have been proposed and evaluated, but they are challenging to implement due to the variety of cells, structure, quality of the image, and resemblance among benign and malignant trials [17,18,19]. In addition, patients with breast cancer who receive an early diagnosis are an important aspect of treatment for decreasing the mortality rate.

Due to the high rate of mortality, new techniques have been developed to increase survival rates through early detection breast cancer [20,21,22]. Recently, many researchers have been working with CADs because they could help doctors make accurate and reliable medical diagnoses [23,24,25,26]. In a study using CADs, Alam et al. removed the triangular location of the pectoral muscle with K-means clustering [27]. Three categories of outcomes from the removal of the pectoral muscle included good at 90.37%, acceptable at 8.07%, and unexpected at 1.5%. Mokni et al. propose a computerized feature categorization of cancer cells using dynamic contrast-enhanced MRI and DMGs [28]. The best area under curve (AUC) values were obtained with the random forest classifier for each modality in the findings of the developed multimodal fusion-based CAD arrangement, which achieved an AUC value of 99.10% using the radial basis function neural network (RBFNN) classifier. Henriksen et al. examined the application of a CAD in mammography screenings [29]. The findings presented that including a CAD improved sensitivity and the rate of cancer detection. Moreover, a new CAD system in digital mammography was presented by Salama et al., using wavelet-based contourlet transform (WBCT) and a hybrid approach to characteristics selection with support vector machines (SVMs), genetic algorithms (GAs), and mutual information (MI) [30]. The combination of WBCT, GA-SVM-MI, and kernel SVM yields the classification accuracy with the highest percentages—97.5% for normal–abnormal and 96% for benign–malignant—although requiring the least resources. Other technologies, like biosensors, can find tumor-associated biomarkers for developing a platform for the primary diagnosis of many cancers [31,32,33]. In a study, Chupradit et al. examine biosensors that are categorized based on transducers, such as piezoelectric, optical, and electrochemical types, to diagnose the human epidermal growth factor receptor 2 (HER-2) [34]. Future studies should focus on developing colorimetric biosensors for HER-2 detection. Salahandish et al. replaced expensive gold nanoparticles with less expensive silver nanoparticles and improved the protocols for the synthesis and functionalization of the nanocomposite in order to create a high-performance biosensing construct made of gold nanoparticle-grafted functionalized graphene and nanostructured polyaniline [35]. Su et al. established the iREX biosensor, capable of quantitatively assessing MUC1, HER2, and CEA in EXO samples [36]. Zheng et al. developed a SERS immunoassay sensor grounded on a microfluidic micro-chip to concurrently diagnose multiple cancer biomarkers in real samples [37]. The outcomes agree with the commercial enzyme-linked immunosorbent assay (ELISA) kits, demonstrating the accuracy of the SERS-based microfluidic immunoassay.

Although traditional CADs and biosensor technologies provide high-quality early cancer detection, they both have significant limitations. The CAD is complex software that requires time and training, making it difficult for those with limited resources [38]. To improve machine learning performance, a CAD necessitates managing a lot of combined training information and computer resources [39]. Moreover, high-quality CAD software can be costly, especially when utilized for viable or professional reasons [40]. This will be the precedent to some malignant lesions being dismissed by endoscopists [41]. Additionally, the presentation of traditional CAD structures is distant from the necessities of instantaneous applications due to limits such as quality of the image, information size, trust of doctors, and regulatory and commercial standards [42]. Most cancer biosensors, on the other hand, have limited reliability due to the biocompatibility of the immobilization matrices employed in developing them. Furthermore, weak biological signals produced by the interaction of biorecognition molecules with biomarkers may reduce detection sensitivity [43,44]. However, hyperspectral imaging (HSI) is a pioneering method that has the capability to overcome all of the challenges. It can improve the cancer detection performance by capturing images across the entire spectrum of light, from ultraviolet (UV) to far-infrared (FIR), instead of just the RGB, providing more detailed information about the subject in a non-invasive manner [45].

**Figure 1:**
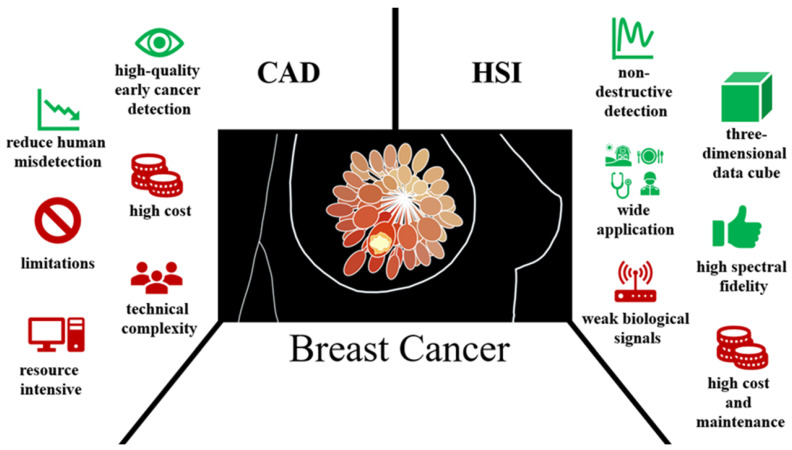
New research on diagnosing breast cancer by combining the advantages and disadvantages of HSI and CAD technology.

HSI is a spectrum-sensing technology that photographs an object applying many definite ocular wavelengths through a wide spectral range [46,47,48]. It is an NDT method that allows it to obtain the most accurate and complete information [49,50]. An HSI image is created by detecting the 2D spectral data of every single pixel and acquiring both spatial and spectral evidence. Consequently, the source of every gamut in the picture is determined [51]. The imaging spectrum was developed in the 1980s and is used for photography in the UV, visible (VIS), and near-infrared (NIR) areas of electromagnetic waves [52]. It can photograph in a wide range of continuous and narrow bands, giving each pixel a completely reflected or emitted spectrum. HSI is a type of imaging that captures data in wavelengths other than the three basic color bands [53]. When compared with RGB data, HSI is more detailed and has better spectral fidelity than RGB data [54,55,56]. It also contains more spectrum information than RGB data, can be utilized to obtain spectral signatures of natural scenes, and can be identified by high-dimensional data and an inadequate amount of training examples [57,58,59,60]. According to the image acquisition process, HSI systems operate in four modes: whiskbroom, pushbroom, liquid crytal tunable filter, and snapshot [61], producing a 3D hypercube with a spectral and two spatial axes. Whisk broom sensors have more moving parts that wear out quicker [62]. Pushbroom cameras contain fewer repositioning components, but nonetheless require consistent standardization because diverse sections may demonstrate different sensitivity, resulting in bands inside the data cube [63]. Tunable filter sensor for one waveband image at a time [64]. The snapshot technique is a way to capture the HSI without scanning, with a single-shot mode requiring no movement of the platform or detector [65]. Figure 1 shows the new research on diagnosing breast cancer by combining the advantages and disadvantages of HSI and CAD technology.

Hyperspectral macroscopic imaging and hyperspectral microscopic imaging markedly differ in their applications and detail resolution, each providing distinct benefits for biological study [66,67]. Hyperspectral macroscopic imaging acquires spectral data over extensive tissue areas, offering an expansive perspective beneficial for analyzing structural patterns, recognizing abnormalities, and pinpointing locations of interest for subsequent examination. This method is optimal for scenarios when doctors want an extensive field of view to identify aberrant tissue at a macroscopic level, such as in surgical guiding or preliminary screening, without examining cellular specifics [68]. Macroscopic imaging is advantageous for non-invasive evaluations, as it rapidly encompasses large tissue regions. Conversely, hyperspectral microscopic imaging emphasizes the acquisition of high-resolution spectral data at the cellular or subcellular level, providing comprehensive insights into the tissue form and biochemical composition [69]. This tiny size is essential for identifying small differences inside cells, such as recognizing early-stage malignancy via molecular markers that may not be discernible at a macroscopic level. It necessitates more advanced equipment and frequently more computer power because of the resolution and data volume. These two imaging scales complement one another, with macroscopic imaging facilitating early detection and microscopic imaging offering detailed diagnostic information, so improving the whole diagnostic process in clinical and research environments. Ex-vivo studies in HSI for medical diagnostics can be broadly categorized into histological studies and preliminary studies intended for in vivo applications, each serving distinct purposes in research and clinical translation [70,71]. Histological ex vivo studies focus on analyzing tissue samples removed from the body to explore cellular and molecular details. These studies often examine the specific spectral signatures of cancerous and non-cancerous tissues at a microscopic level, yielding high-resolution data on tissue characteristics. This controlled approach is valuable for understanding cancer pathology and validating hyperspectral imaging’s capacity to differentiate tissue types. Conversely, preliminary ex vivo studies for in vivo applications simulate live conditions to assess HSI methods’ practical feasibility before deploying them in real-time patient care. These studies often prioritize factors such as speed, non-invasiveness, and broader tissue coverage, aiming to develop HSI techniques that can transition smoothly into in vivo settings. For instance, testing HSI on freshly excised tissues allows researchers to refine imaging protocols and optimize algorithms for live diagnostic use.

Significant advancements in HSI have occurred over the past three decades, resulting in its emergence as a useful tool. It is becoming increasingly popular for remote detection functions such as agriculture [72,73], the environment [74,75], air pollution detection [76,77], food analysis [78,79], biomedical [80,81], military [82,83], counterfeit applications [84,85], management of natural resources [86,87], aerospace [88,89], detecting natural disasters [90,91,92], and more [93,94,95,96]. HSI has been exploited extensively in the diagnosis of cancer, including brain cancer [97,98,99,100], esophageal cancer [101,102,103], lung cancer [104,105,106], prostate cancer [107,108,109], gastric cancer [110,111,112], skin cancer [113,114,115], and breast cancer [116,117].

This article explores current studies on the detection and categorization of breast cancer using the HSI method in amalgamation with CAD approaches. The study assesses the precision of the CAD in combination with HSI algorithms for the prognosis and detection of breast cancer. It assesses the diagnostic capabilities using metrics of sensitivity, specificity, accuracy, and area under the curve (AUC). The evaluation presents a brief summary of the study and provides commendations dependent on the meta-analysis of different CAD + HSI methods used.

## 2. QUADAS-2 Assessment

### 2.1. Study Selection Criteria

The reason for this study is to clarify the advancements made in the identification and prognosis of breast cancer by means of HSI, while also discussing the gains and drawbacks of the system. This review specifically evaluates studies that satisfy the subsequent criteria for inclusion: The study’s inclusion criteria are as follows: (1) studies that present precise quantitative information, such as dataset, precision, and recall; (2) studies that concentrate on the identification of breast cancer using HSI; (3) manuscripts with results published within the past eight years; (4) studies that utilize a combination of prospective and retrospective proposals; and (5) research studies that are written in English (see Supplement Figure S1 for the search process flowchart;). Additionally, this assessment eliminates research that fulfills the specified criteria for exclusion, which encompass: (1) manuscripts without sufficient data; (2) narrative, systematic assessment, and meta-analysis studies; (3) comments, proceedings, or study procedures; and (4) manuscripts presented at conferences. The authors (P.T. and H.-C.W.) employed the Quality Assessment of Diagnostic Accuracy Studies Version 2 (QUADAS-2) to scrutinize the methodology of the publications being evaluated in this study [118,119,120]. The participant screening and index test in QUADAS-2 incorporate bias assessments. Furthermore, it assesses the level of accuracy and the potential for skew in terms of time and flow as areas of focus [121].

### 2.2. QUADAS-2 Results

The QUADAS-2 findings from the eight pieces of research analyzed in this study are outlined in Table 1. The text examines the concerns of relevance and the extent of prejudice in the research. Each study had a comprehensive evaluation to assess patient selection, index test, reference standard, bias risk, and application concerns, including flow and timing (see Supplement Figure S2 for QUADAS-2 Domain).

## 3. Results

This part provides an exposition of the findings from the review, encompassing the clinical characteristics that were noticed and a comprehensive summary of every piece of research. Additionally, this segment contains the numerical conclusions of every item of research. The section additionally examines the manner in which the outcomes were evaluated with respect to the degree of precision, recall, and accuracy.

### 3.1. Studies under Clinical Feature Observation

Aboughaleb et al. employed HSI in conjunction with K-means clustering to examine the data from 10 patients with ex vivo breast cancer. These individuals were undergoing breast malignancy and had undergone a process of eradication [122]. The results indicate that using superficial spectral reflection imaging at a wavelength of 500 nm may effectively differentiate between cancerous and normal tissues. Furthermore, the HSI system established a superior level of accuracy in distinguishing the tumor region from regular tissue, by a sensitivity of 95% and specificity of 96%. Kho et al. employed HSI alongside two distinct classification methods, namely U-Net and LDA spectral classification algorithms. The study involved 29 patients who underwent primary breast surgery, and aimed to detect tumors on fresh ex vivo tissue. The objective was to regulate the wavelength range which yielded the maximum effective discrimination between cancers and healthy breast tissue [123]. Each tissue slice comprises both malignant and non-malignant tissue. All of the high-resolution photos were tagged with four tissue categories: ductal carcinoma in situ (DCIS), invasive carcinomas (ICs), connective tissue, and adipose tissue. The data indicate that utilizing mutually the visible (VIS) and near-infrared (NIR) wavelength bands yielded the maximum recall rates for both IC and DCIS. In addition, U-net has superior sensitivity and specificity compared to LDA, with respective values of 0.80, 0.93, 0.76, and 0.92.

Jong et al. devised three distinct convolutional neural networks (CNNs): the One-dimensional Convolutional Neural Network (1D-CNN), the Depthwise Convolutional Neural Network (DC-CNN), and the Three-dimensional Convolutional Neural Network (3D-CNN). The categorization of normal tissue from cancerous tissue in lumpectomy samples from removed breast samples from 29 patients with principal breast cancer was performed using an evenly distributed categorical cross-entropy (BCCE) loss function and a pixel-distance-excluding (PDE) loss function. The results indicate that the DC-CNN achieved the best rate of classification for both loss functions [124]. Ortega Sarmiento et al. employed the whole of 112 hyperspectral pictures, with 65 originating from Patient 1 and 47 from Patient 2, for the analysis of histology samples. They employed HSI and deep learning techniques to develop a personalized 2D-CNN for the automated distinction of malignancy cells from normal breast cells. Next, the classification of HSI and traditional Red Green Blue (RGB) images was compared. The findings indicated that the accuracy is 84%, the sensitivity rate is 83%, and the specificity rate is 85% [125].

Khouj et al. conducted an analysis to assess the effectiveness of a snapshot hyperspectral imager in identifying spectral variations between normal and cancerous breast tissues. The study involved 10 patients, with samples consisting of both normal and DCIS tissue. Image processing systems were employed by means of both supervised and unsupervised data [126]. Findings demonstrate the successful identification of spectral tissue variations, achieving a sensitivity of 85.45% and a specificity of 94.64%. Aref et al. introduced an optical imaging module with a HS camera that can accurately identify and detect the boundaries of breast cancer in 30 patients. The system utilizes the HSI module in the VIS-NIR region to analyze both standard and DCIS tissue samples. In addition, it utilizes a customized K-mean clustering technique and contour delineation to recognize the specific regions in the breast cancer locations. The findings indicate that the wavelength most commonly associated with higher Rd intensity and the ability to accurately determine the location of the tumor was 447 nm. Additional specimens exhibited benign characteristics at a wavelength of 524.35 nm. In addition, this method had a sensitivity of 98.95% and a specificity of 98.44%. It can help the physician and diagnostician differentiate between tumorous tissue and non-tumorous tissue quickly and without the need for intrusive procedures, saving time [127].

Wang et al. developed a U-Net model called PCA-U-Net using principal component analysis to distinguish between malignant breast cells and normal tissues. The model was trained using samples obtained from 30 patients, varying in age from 22 to 82 years [128]. The PCA-U-Net technique achieved an accuracy of 87.14%, a sensitivity of 80.51%, and a specificity of 84.1% according to the results. Pathologists might utilize it to assist them in formulating a pathological finding of breast cancer. Moreover, the technique decreases the duration needed for the preparation of samples, data collection, and image processing, hence enhancing diagnostic efficiency. Kho et al. obtained data from tissue slices of 18 patients following the removal of the breast specimen. The information was separated into two datasets: HS and lumpectomy. The provided information was utilized to develop and evaluate an SVM classification method. Additionally, an investigation was conducted to establish whether HSI has the capability to identify cancers, such as DCIS and IC, on the resection surface. The findings demonstrate that HSI had a notable diagnostic efficacy when applied to tissue sections. The system of classification that was created yielded the following results: the estimated accuracy, sensitivity, and specificity of IC were 93%, 94%, and 94% respectively, though for DCIS they were 85%, 96%, and 96%, respectively, with a total of 183 cases [129].

Table 2 shows the observed studies under clinical features. These studies involve different HSI methods, wavelength bands, and nationalities for discriminating tumor tissue from breast cancer patients [122,123,124,125,126,127,128,129]. Most of these studies used a Western sample, but only one out of eight studies used a sample from Asia [128]. Three studies used K-mean clustering [122,126,127], and two studies used CNN [124,125], SNV [123], PCA [128], and SVM [129]. In the case of the wavelength range band to distinguish between malignant and non-cancerous tissue, four out of eight studies used the VIS and NIR ranges [123,124,125,127], two out of eight used the VIS range [122,126], one study used the NIR range [129], and one study used a combination of all the three bands (UV + VIS + NIR) [128].

### 3.2. Meta-Analysis of the Studies

The breast cancer subcategory and diagnostic test accuracy (DTA) meta-analysis are shown in Table 3. In this review, the averages of accuracy, specificity, and sensitivity from eight studies were 87% (80% to 93%), 89% (36% to 98%), and 78% (62% to 99%), respectively. Seven of the eight studies in the study were from the West, which has a high sensitivity of 77.39%, a specificity of 89.08%, and an accuracy of 86.93%. The others came from Asia and have a high sensitivity of 84.12%, a high specificity of 84.12%, and a high accuracy of 87.14%. Although Asian studies have a higher sensitivity and accuracy than Western studies, there was only one Asian study, so it was impossible to compare or confirm their performance.

Ten of the fifteen methods used CNN and K-mean clustering, with CNN having a sensitivity of 65.43%, a specificity of 83.29%, and an accuracy of 87.43%. Another three studies used K-mean clustering, which has a sensitivity of 93.13%, a specificity of 96.36%, and an accuracy of 80.27%. SVM, on the other hand, was used in 2 of the 15 methods and achieved the highest sensitivity, specificity, and accuracy of 95%, 95%, and 88.50%, respectively, although the SVM had the highest precision in terms of sensitivity, specificity, and accuracy. For statistical analysis, more studies are still needed.

Many studies used different wavelength bands to distinguish tumors from normal tissue. As the results show, using a single wavelength achieves better sensitivity, specificity, and accuracy data than using multiple wavelengths, such as in the NIR, where sensitivity is 95%, specificity is 95%, and accuracy is 88.50%. Similarly, in the VIS, sensitivity is 90.23%, specificity is 95.32%, and accuracy is 80.27%. However, the most common is in the visible and VIS + NIR ranges; based on studies, 10 out of 15 use these ranges, which have a sensitivity of 71.30%, a specificity of 86.64%, and an accuracy of 87.43%. A combination of all three bands (UV + VIS + NIR) has a higher sensitivity of 84.12% than the VIS + NIR ranges. However, even using wavelengths in the VIS, NIR, and UV + VIS + NIR performed well, but only in limited quantities, limiting the sample size for the analysis. More research is required for statistical analysis.

As can be seen, the sensitivity and accuracy for studies published before 2019 are 85.45% and 80.27%, respectively, which are lower than the sensitivity and accuracy for studies published between 2019 and 2021, which are 88.16% and 88.04%. These can be explained by the fact that the HSI technique is constantly evolving year after year.

### 3.3. Meta-Analysis of Subgroup

**Figure 2:**
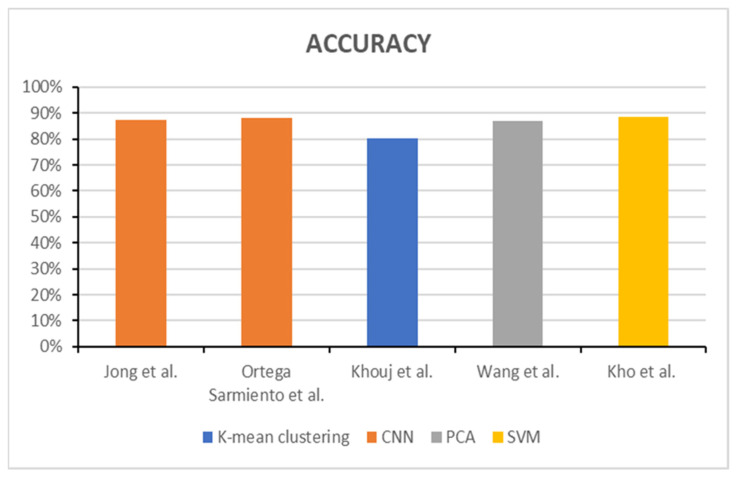
Overall accuracy chart for each HSI method (Jong et al./2022 [124], Ortega et al./2020 [125], Khouj et al./2018 [126], Wang et al./2021 [128], Kho et al./2019 [129]).

The accuracy chart below compares the HSI approaches utilized in the research grounded on diagnosis and detection of breast cancer. Figure 2 represents the total accuracy of each of the different HSI methods. CNN was the most used HSI method. However, the greatest accuracy of 88.5% was obtained by using the SVM method in the study of Kho et al. Nevertheless, studies using K-mean clustering by Aboughaleb et al. [122] and Aref et al. [127], as well as SNV methods by Kho et al. [129] have no accuracy published in the research.

**Figure 3:**
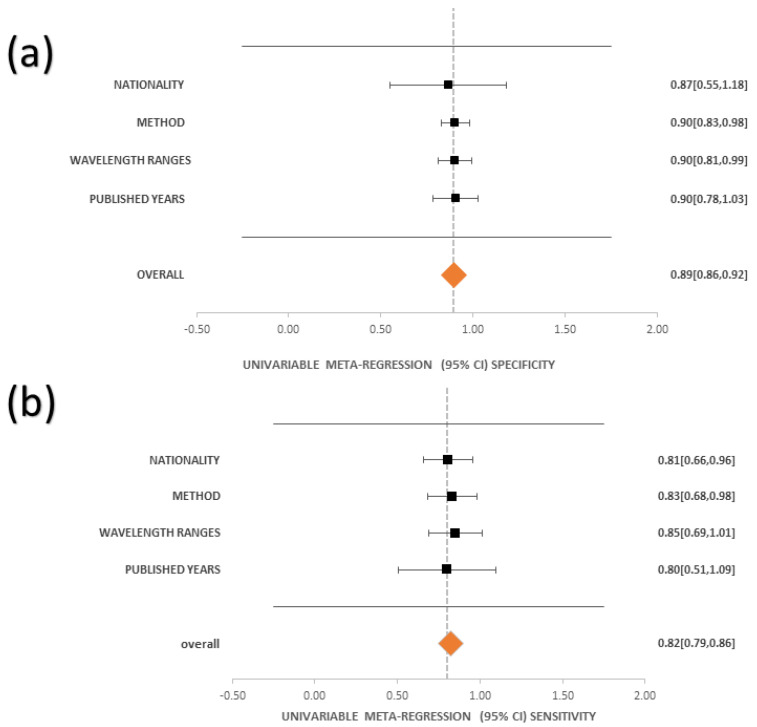
The univariable meta-regression of (**a**) specificity and (**b**) sensitivityat a 95% CI.

Moreover, the forest plot was designed to interpret the sensitivity and specificity of each study, which were produced with a 95% level of confidence. According to the study classification, in the sensitivity forest plot, studies by Kho et al. [129] that used SNV and Jong et al. [124] that used CNN methods are not statistically significant because the confidence interval overlaps with the null line. In contrast, studies by Kho et al. [129] that used SVM methods are statistically substantial because the confidence interval does not overlay with the null line. Furthermore, the meta-regression examination was designed to interpret the sensitivity and specificity of the classification data based on nationality, method, wavelength ranges, and published years. Figure 3 presents the univariable meta-regression of sensitivity and specificity at a 95% confidence level (see Supplement Figures S3 and S4 for Sensitivity and Specificity Forest Plot of All Classification and Studies respectively). As the results show, based on wavelength ranges, the best performance was under the meta-regression sensitivity with 0.85 (0.69, 1.01), and based on HSI methods, the best performance was under the meta-regression specificity with 0.9 (0.83, 0.98) (Tables S1 and S2 shows the Sensitivity and Specificity Computations for Forest Plot HSI Method and Studies while Table S3 shows the Sensitivity and Specificity Computations for Meta Regression).

**Figure 4:**
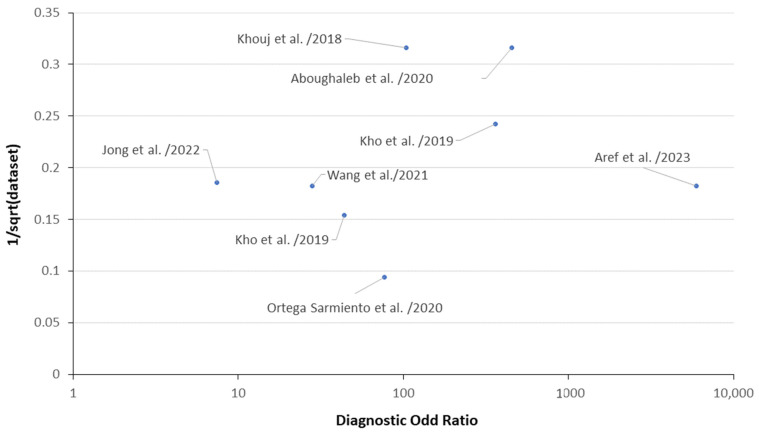
Deeks’ funnel plot of studies (Aboughaleb et al./2020 [122], Kho et al./2019 [123], Jong et al./2022 [124], Ortega et al./2020 [125], Khouj et al./2018 [126], Aref et al./2023 [127], Wang et al./2021 [128], Kho et al./2019 [129]).

Finally, Deeks’ funnel plots are simple scatterplots showing every study (x-axis) versus a metric of study size (y-axis) [130,131] (see Supplement Figures S5–S8 for the Deeks’ Funnel Plot for Nationality, Methods, Wavelength Ranges and published years). They were created with classifications of nationality, HSI methods, wavelength ranges, published years, and in all classifications (Tables S4–S7 shows the Regression Statistics of Studies, Wavelength ranges, Nationality and HSI Methods respectively). The results show a *p*-value of 0.89 for nationality, 0.15 for HSI methods, 0.11 for wavelength ranges, 0.29 for published years, and 0.87 for all classifications. This means that Deeks’ funnel plots created from studies show no indication of heterogeneity. Figure 4 describes the Deeks’ funnel plots of the research (Tables S8–S13 shows the p-value of Deeks’ Funnel Plot Studies, Nationality, HSI Methods, Wavelength ranges, Published Years and Classification respectively.). All of these studies were correlated.

## 4. Discussion

Currently, ML techniques have aided pathologists in routine sample examination by providing quantitative disease diagnosis and decreasing inter-observer variability [132]. An advanced HSI system uses an endoscope and liquid crystal tunable filter technology to detect cancer [133]. This technology enables spatially accurate images of cancerous versus healthy tissues, which may improve human optical diagnosis. This review discussed a combination of CADs and HSI to detect breast cancer. Several recent studies into cancer detection using HSI technology have been published, with excellent results as computer-based auxiliaries in the analysis and identification [134]. CNN, SVM, and K-means clustering were extensively utilized owing to their unique methodologies and common application in the HSI literature for medical diagnosis. Each technique possesses distinct advantages for breast cancer classification: CNNs are esteemed for their exceptional efficacy in image classification, utilizing spatial patterns in hyperspectral data; SVMs are acknowledged for their resilience in managing intricate, high-dimensional datasets like HSI, demonstrating robust performance on comparatively smaller datasets; K-means clustering serves as a potent unsupervised technique that can elucidate spectral distinctions between malignant and benign tissues without the necessity for extensive labeled data, which is often scarce in medical imaging.

Even though HSI technology has a good ability to diagnose and detect, in scientific exercise, breast cancer management and diagnosis are still determined by the endoscopist. The use of HSI technology in breast cancer diagnosis will become more acceptable. The review demonstrated the competence of HSI technology for primary breast cancer detection. Several studies in this review focused on distinguishing malignant tissue from normal tissue in early-stage breast cancer and produced impressive results. The possibility of diagnosing breast cancer will provide information for early treatment and improve future performance.

Despite its excellent diagnostic performance, the study had several limitations. Firstlyits patient limit was 280, which was lower than the other methods. For the reason that this limitation may have impacted the results of the studies involved, it is best to consider recruiting more participants. Secondly, as identified in this review, the limited nationality data included only one study from Asia. Expanding the sample size and including a more diversified geographical dataset, especially with supplementary research from Asia, might substantially strengthen the validity of the findings. Diverse geographical data can elucidate variances in breast cancer presentations among populations, encompassing disparities attributable to genetic, environmental, and lifestyle variables. An extended dataset may uncover distinct patterns or effectiveness variations in HSI diagnostic procedures among ethnic groups, thereby producing more tailored or culturally attuned diagnosis methodologies. Thus, the performance of the Asian study cannot be considered for statistical analysis. Furthermore, due to limitations in the classification of differences using the methods, the CNN method was the most popular in this review, as was K-mean clustering, which provided a high level of sensitivity and specificity for the finding and diagnosis. Other methods, such as SNV, PCA, and SVM, also performed well, but only in a limited quantity, making the sample size for the statistical analysis small. Multi-wavelength ranges (VIS + NIR, UV + VIS + NIR) were used more in this review than single-wavelength ranges (VIS, NIR). In comparison, using a single wavelength range provided better execution in the relationship with sensitivity and specificity than using a multi-wavelength range, but this cannot be determined to compare because of the minimum amount of research that uses a single wavelength range. The discrepancies in wavelength selection among studies might impact the generalizability of our findings, since each decision may change the sensitivity and specificity in differentiating malignant from healthy tissues. Future studies aimed at standardizing wavelength ranges and identifying ideal spectral bands for breast cancer detection would significantly enhance the consistency and comparability of HSI results across various clinical environments. This domain warrants more inquiry to enhance HSI methodologies, rendering them more generally applicable and successful across varied populations. Finally, due to the publication year limitations, the number of studies published before 2019 and studies published in 2022–2023 is limited, making it impossible to consider the development of HSI technology. The increase in sensitivity and accuracy in research published from 2019 to 2021 certainly indicates substantial progress in both hardware and analytical methodologies in HSI diagnosis. Recent advancements in HSI sensor technology have enabled improved spectral resolution and faster data gathering, hence immediately enhancing the detail and quality of tissue distinction. Furthermore, advanced algorithms—such as enhanced CNN architectures and improved machine learning classifiers—have been created, more effectively capturing the intricate spectral fingerprints of malignant and benign tissues.

However, these studies possess specific limitations that necessitate evaluation. The limited sample size may undermine the validity of our results; hence, future research may benefit from bigger, multi-center datasets to improve generalizability. Incorporating varied populations into databases may mitigate biases and reflect variability in breast cancer presentations among different demographics. Secondly, discrepancies in methodology and wavelength ranges across the included research hindered direct comparisons, highlighting the necessity for uniform protocols in HSI data gathering and diagnostic criteria. Standardizing these aspects will enhance the consistency of cross-study analyses and augment dependability. Ultimately, the advancement in HSI technology and machine learning algorithms specifically designed for hyperspectral data processing might markedly improve diagnosis accuracy. Incorporating these guidelines in future studies may alleviate these limitations and establish a more robust basis for assessing the clinical viability of HSI in breast cancer detection. Integrating HSI with CAD systems has significant diagnostic potential, but several challenges that could affect its adoption in routine clinical workflows. One major obstacle is the high cost and complexity of HSI equipment, which may limit its accessibility in some healthcare settings. Additionally, HSI generates large data volumes, requiring robust data storage and processing capabilities and specialized training for clinical staff to interpret results effectively. To overcome these challenges, streamlined, user-friendly CAD interfaces and AI-driven data processing algorithms can help integrate HSI more seamlessly into existing workflows. Developing cost-effective, portable HSI systems could also make this technology more accessible. Furthermore, offering training programs for clinicians and radiologists will be essential to familiarize them with HSI data interpretation. With these strategies, we believe HSI–CAD integration could become a more feasible, impactful tool in breast cancer diagnostics. Perfusion oncologic HSI is an advanced non-invasive imaging technique used to assess tissue perfusion and vascular characteristics in cancerous tissues [135]. It leverages the unique ability of HSI to capture detailed spectral information across multiple wavelengths, allowing for the detection of subtle changes in tissue composition, including blood flow, oxygenation, and other metabolic processes crucial in oncologic diagnostics [136].

## 5. Conclusions

This paper examined the performance of HSI in detecting breast cancer. Several features and classifiers were investigated. Study results show that combining CADs and HSI has the potential to greatly advance the precision and competence of breast cancer diagnosis. Combining CAD methods with HSI has been demonstrated to be advantageous for the diagnosis of breast tumors in this study. Moreover, the results were statistically insignificant, and there was no heterogeneity among these studies. The effectiveness of HSI is determined by classification, such as the type of wavelength band used for distinguishing between malignant and normal tissues, as well as the type of algorithm used. Furthermore, particular features of investigation and research should be well thought out, such as identifying the restrictions of the studies involved, the number of breast cancer patients, methods used, and wavelength bands used. Further research and development in technology will be invaluable in achieving the full potential of HSI for breast cancer detection and, ultimately, improving patient outcomes worldwide.

## Figures and Tables

**Table 1:** QUADAS-2 Summary.

Study	Risk of Bias				Applicability Concerns		
	Patient Selection	Index Test	Reference Standard	Flow and Timing	Patient Selection	Index Test	Reference Standard
Aboughaleb et al./2020 [122]	+	+	+	+	+	+	+
Kho et al./2019 [123]	+	+	+	+	+	+	+
Jong et al./2022 [124]	+	?	+	+	+	?	+
Ortega et al./2020 [125]	+	?	+	+	+	?	+
Khouj et al./2018 [126]	+	+	+	+	+	+	+
Aref et al./2023 [127]	+	+	+	+	+	+	+
Wang et al./2021 [128]	+	+	?	?	+	+	+
Kho et al./2019 [129]	+	+	+	+	+	+	+

+ Low Risk, − High Risk, ? Unclear Risk.

**Table 2:** Studies under Clinical Feature Observation.

Study	Nationality	Index Number	Method	Numberof Patients	Sensitivity (%)	Specificity (%)	Accuracy (%)	Band
Aboughaleb et al./2020 [122]	Western	1	K-mean	10	95.00	96.00	NA	VIS
Kho et al./2019 [123]	Western	2	LDA + SNV	42	76.00	92.00	NA	VIS + NIR
		3	U-NET + SNV	42	80.00	93.00		
Jong et al./2022 [124]	Western	4	BCCE + 1D-CNN	29	67.00	97.00	92.00	VIS + NIR
		5	BCCE + DC-CNN		62.00	95.00	89.00	
		6	BCCE + 3D-CNN		0.00	36.00	80.00	
		7	PDE + 1D-CNN		87.00	90.00	90.00	
		8	PDE + DC-CNN		78.00	94.00	91.00	
		9	PDE + 3D-CNN		72.00	84.00	82.00	
Ortega et al./2020 [125]	Western	10	2D-CNN	112	92.00	87.00	88.00	VIS + NIR
Khouj et al./2018 [126]	Western	11	K-means	10	85.45	94.64	80.27	VIS
Aref et al./2023 [127]	Western	12	K-means	30	98.95	98.44	NA	VIS + NIR
Wang et al./2021 [128]	Asian	13	PCA + U-Net	30	84.12	84.12	87.14	UV + VIS + NIR
Kho et al./2019 [129]	Western	14	SVM	8	94.00	94.00	93.00	NIR
		15		9	96.00	96.00	84.00	

NA—Not Available.

**Table 3:** Subgroup and Diagnostic Test Accuracy Meta-analysis.

Subgroup	Number of Studies	Sensitivity (%)	Specificity (%)	Accuracy (%)
Average meta-analysis	8	77.83	88.75	86.95
Nationality				
Western	7	77.39	89.08	86.93
Asian	1	84.12	84.12	87.14
Methods				
K-mean	3	93.13	96.36	80.27
CNN	7	65.43	83.29	87.43
SNV	2	78.00	92.50	NA
PCA	1	84.12	84.12	87.14
SVM	2	95.00	95.00	88.50
Wavelength range bands				
VIS + NIR	10	71.30	86.64	87.43
VIS	2	90.23	95.32	80.27
NIR	2	95.00	95.00	88.50
UV + VIS + NIR	1	84.12	84.12	87.14
Published Years				
Before 2019	1	85.45	94.64	80.27
2019–2021	5	88.16	91.73	88.04
2022–2023	2	66.42	84.92	87.33

NA—Not Available.

## Data Availability

The data presented in this study are available in this article upon request to the corresponding author (H.-C.W.).

## Supplementary Materials

The following supporting information can be downloaded at: https://www.mdpi.com/article/10.3390/bioengineering11111060/s1, Figure S1. Search Process Flowchart; Figure S2. QUADAS-2 Domain; Figure S3. Sensitivity and Specificity Forest Plot (All Classification); Figure S4. Sensitivity and Specificity Forest Plot (Studies); Figure S5. Deeks’ Funnel Plot for Nationality; Figure S6. Deeks’ Funnel Plot for HSI Methods; Figure S7. Deeks’ Funnel Plot for Wavelength Ranges; Figure S8. Deeks’ Funnel Plot for Published years; Table S1. Sensitivity and Specificity Computations for Forest Plot (HSI Method); Table S2. Sensitivity and Specificity Computations for Forest Plot (Studies); Table S3. Sensitivity and Specificity Computations for Meta Regression; Table S4. Regression Statistics (Studies); Table S5. Regression Statistics (Nationality); Table S6. Regression Statistics (HSI Methods); Table S7. Regression Statistics (Wavelength ranges); Table S8. *p*-value of Deeks’ Funnel Plot (Studies); Table S9. *p*-value of Deeks’ Funnel Plot (Nationality); Table S10. *p*-value of Deeks’ Funnel Plot (HSI Methods); Table S11. *p*-value of Deeks’ Funnel Plot (Wavelength ranges); Table S12. *p*-value of Deeks’ Funnel Plot (Published Years); Table S13. *p*-value of Deeks’ Funnel Plot (Classification).
