# Supplementary material for: Systematic Meta-Analysis of Computer-Aided Detection of Breast Cancer Using Hyperspectral Imaging
Source: Bioengineering (Basel). 2024 Oct 24;11(11):1060. doi: 10.3390/bioengineering11111060 (PMC11591395; doi:10.3390/bioengineering11111060)
Supplement: Supplementary file 1 [file bioengineering-11-01060-s001.zip › bioengineering-3250649-supplementary.pdf]

Review

Systematic Meta-Analysis of Computer-Aided Detection of Breast Cancer Detection using Hyperspectral Imaging: Supplementary Material

Joseph-Hang Leung [1], Riya Karmakar [2], Arvind Mukundan [2], Pacharasak Thongsit [3], Meei-Maan Chen [4], Wen-Yen Chang [5,*], and Hsiang-Chen Wang [2,6,7]

1. Department of Radiology, Ditmanson Medical Foundation Chia-yi Christian Hospital, Chia-yi City, Taiwan; 01289@cych.org.tw (J.H.L)
2. Department of Mechanical Engineering, National Chung Cheng University, 168, University Rd., Min Hsiung, Chia Yi 62102, Taiwan; d09420003@ccu.edu.tw (A.M.); karmakarriya345@gmail.com (R.K.)
3. Faculty of Mechanical Engineering, King Mongkut's University of Technology North Bangkok, Pracharat 1 Road, Wongsawang, Bangsue, Bangkok 10800, Thailand; ppacharasak18@gmail.com (P.T)
4. Center for Innovative Research on Aging Society (CIRAS), National Chung Cheng University, 168, University Rd., Min Hsiung, Chia Yi 62102, Taiwan; laicmm@ccu.edu.tw (M.M.C.)
5. Department of General Surgery, Kaohsiung Armed Forces General Hospital, 2, Zhongzheng 1st.Rd., Lingya District, Kaohsiung City 80284, Taiwan; wenyen85@gmail.com (W.-Y.C.)
6. Department of Medical Research, Dalin Tzu Chi Hospital, Buddhist Tzu Chi Medical Foundation, No. 2, Minsheng Road, Dalin, Chiayi, 62247 Taiwan
7. Director of Technology Development, Hitspectra Intelligent Technology Co., Ltd., 4F., No. 2, Fuxing 4th Rd., Qianzhen Dist., Kaohsiung City 80661, Taiwan

* Correspondence: wenyen85@gmail.com (W.-Y.C.) and hcwang@ccu.edu.tw (H.-C.W.)

Abstract: This article provides the supplementary article for the manuscript, "Systematic Meta-Analysis of Computer-Aided Detection of Breast Cancer Detection using Hyperspectral Imaging".

Keywords: Hyperspectral Imaging; Breast Cancer; Computer-Aided Detection; Systematic Meta-Analysis; Deeks' funnel chart; Diagnostic Test Accuracy; Forest charts

S1. Literature Search

Articles used in this study were independently searched by two authors on the web specifically, in the Google Scholar search engine. Literature from recent years (2016-2023) was selected for this review. Any article duplications were disregarded. The process of reviewing the titles and abstracts of the identified articles was necessary to avoid the inclusion of articles irrelevant to the purpose of this review. Furthermore, full-text reviews were done to be able to determine whether the articles met the criteria for inclusion.

S1.1 Inclusion Criteria

This review intends to focus on studies by the established inclusion criteria:

(1) studies should have definitive numerical results such as dataset, sensitivity, specificity, and accuracy.

(2) based on hyperspectral imaging dealing with breast cancer detection.

(3) must be published in the last 8 years.

(4) studies that have a prospective or retrospective design.

(5) studies written in English.

S1.2 Exclusion Criteria

This review will disregard studies that will fall under the following exclusion criteria:

(1) studies with insufficient data.

(2) studies under narrative, systematic review, and meta-analyses.

(3) comments, proceedings, or study protocols.

(4) conference papers.

S1.3 Data Extraction, Primary Outcomes, and Additional Analyses

The extraction and cross-checking of the data were done by two authors (P.T. and A.M). The primary means of communication for data inquiries and validation was through email. The process of synthesizing each study was generated by a diagnostic test accuracy (DTA) and a systematic review process consequentially. Data gathered in the meta-analyses were mostly about accuracy, sensitivity, and specificity of the diagnostic performance based on hyperspectral imaging in each study.

Furthermore, as subgroup analysis composed of the origin of data was recorded geographically. The type of Nationality, type of Methods, type of Wavelength bands, and type of Published years was as well provided in the subgroup table for further analysis.

S1.4 Study Inclusion

A total of 7,040 results were distinguished upon searching in Google Scholar and 2 additional records were considered through thoroughly searching. 1,740 articles were excluded after considering the years these articles were published since this review will solely focus on articles published in recent years (2016-2023). Articles with full-text access were also observed making up about 997 articles considered to be excluded. A total of 4,303 articles were left to be reviewed. Among these 4,295 records were articles with incomplete data, narrative reviews and meta-analyses , comments, and conference papers which are part of the exclusion criteria. Eventually, 8 studies were included in this review. Supplementary figure 1 shows the flowchart of the selection process.

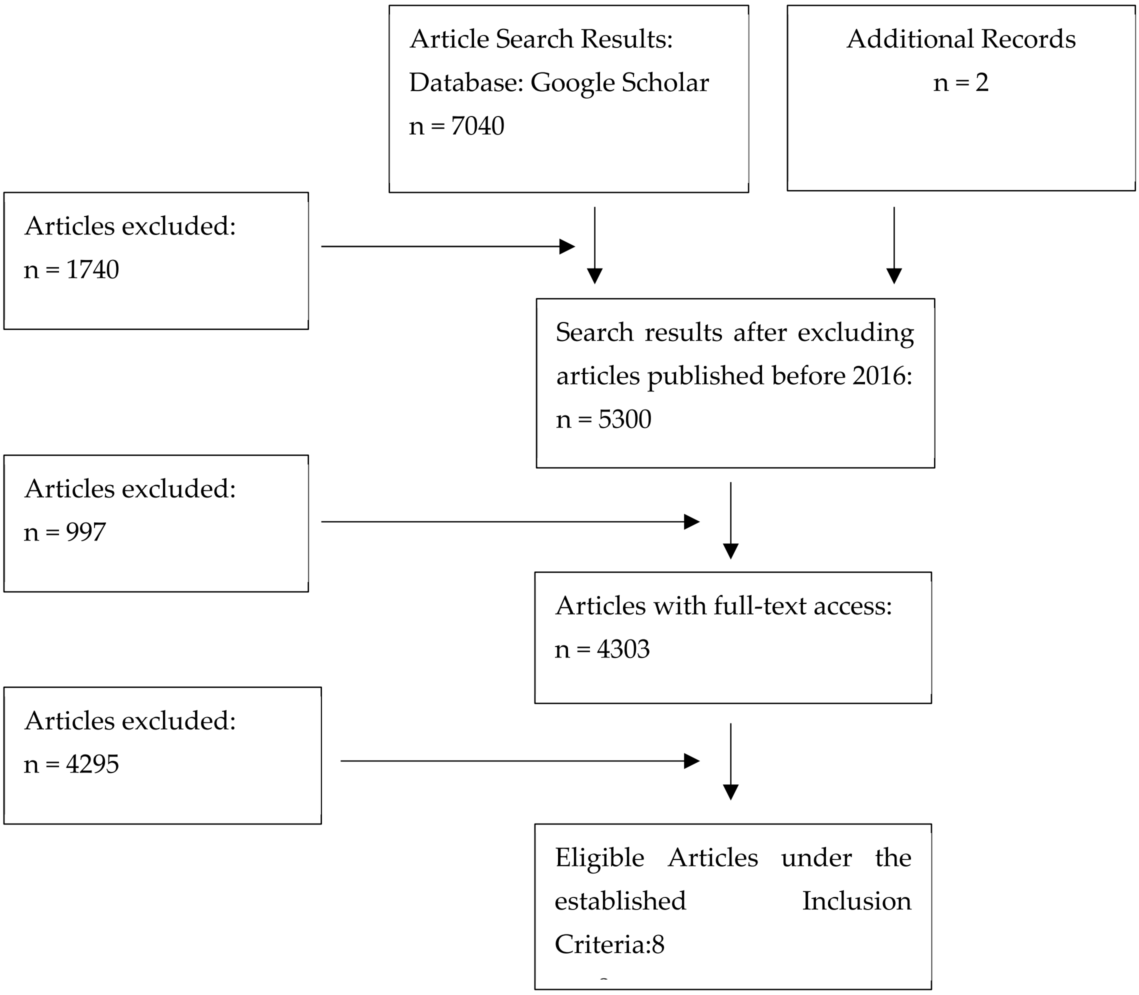

Figure S1. Search Process Flowchart

S2. Quality Analysis

A precise and detailed information from a study to be reviewed is essential and considered to be a good quality for a precise inference. This precise conclusion improves methods in data training and learning. In this study, a couple of breast cancer lesion images and involved breast cancer patients were the main contributor of data needed for training. Nonetheless, risk of bias and concerns regarding applicability must be used since not all studies in this review provided detailed description of the patient enrollment standard, index test, and reference standard. All studies were in "low risk" in terms of concerns regarding applicability. Jong et al., Ortega Samiento et al., and Kho et al. were labelled as "unclear risk" in terms of index test due to the absence of primary outcomes as well as values for specificity and sensitivity. Study by Wang et al. received "unclear risk" in reference standard and flow and timing due to the lack of supporting data for their stated average consistency in diagnostic accuracy.

S2.2 QUADAS-2

This section summarizes the QUADAS-2 results from the eight studies included in this review. It includes concerns about applicability and the level of bias in the studies based on flow and timing, patient selection, reference standard, and index test. Each study was reviewed for bias risk under flow and timing, patient selection, reference standard, and index test, as well as applicability concerns under patient selection, reference standard, and index test.

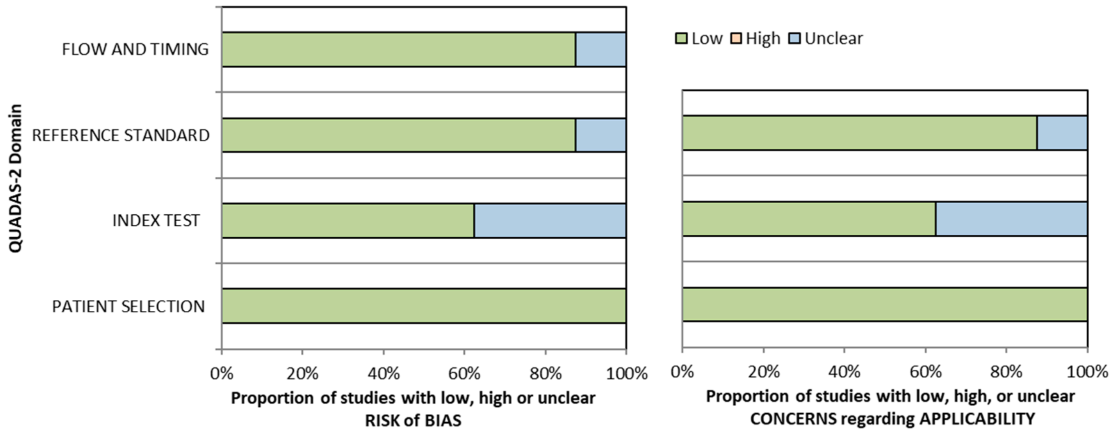

Figure S2. QUADAS-2 Domain

S3. Forest Plot

This section contains sensitivity and specificity forest plots for various classifications relevant to this study, such as the studies involved, the method, and the wavelength band. The forest plot explains the data quality involved in each classification at the 95% level of confidence, as well as its upper and lower limits. The line of null effect can be used to interpret the quality of data from the forest plot. In this study, the line of null effect was calculated by averaging the sensitivity and specificity of the data involved in each classification. The data that overlaps the null effect line is described as low-performance data. In contrast, the data without overlapping the null effect line infers the high performance of the data.

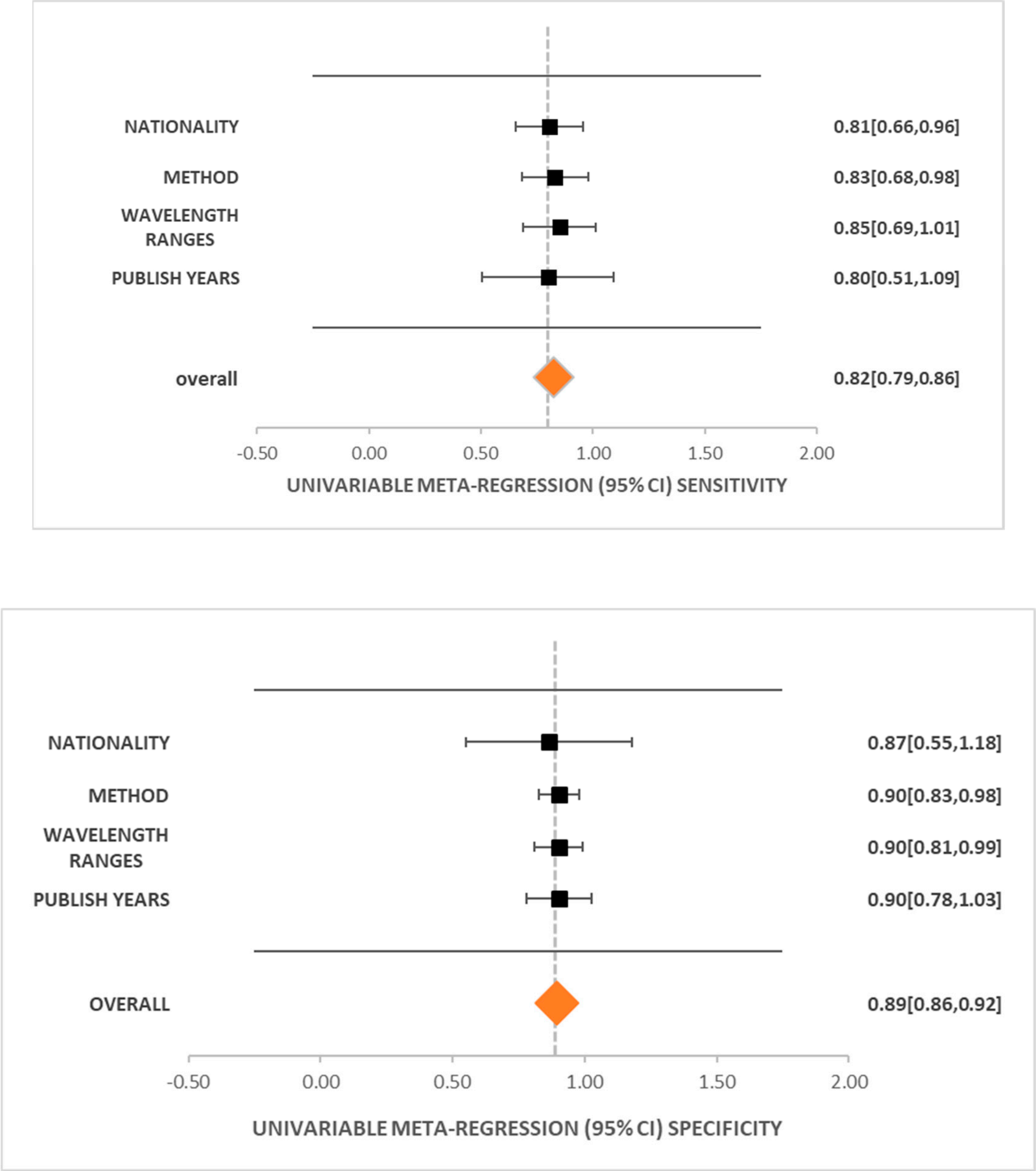

Figure S3. Sensitivity and Specificity Forest Plot (All Classification)

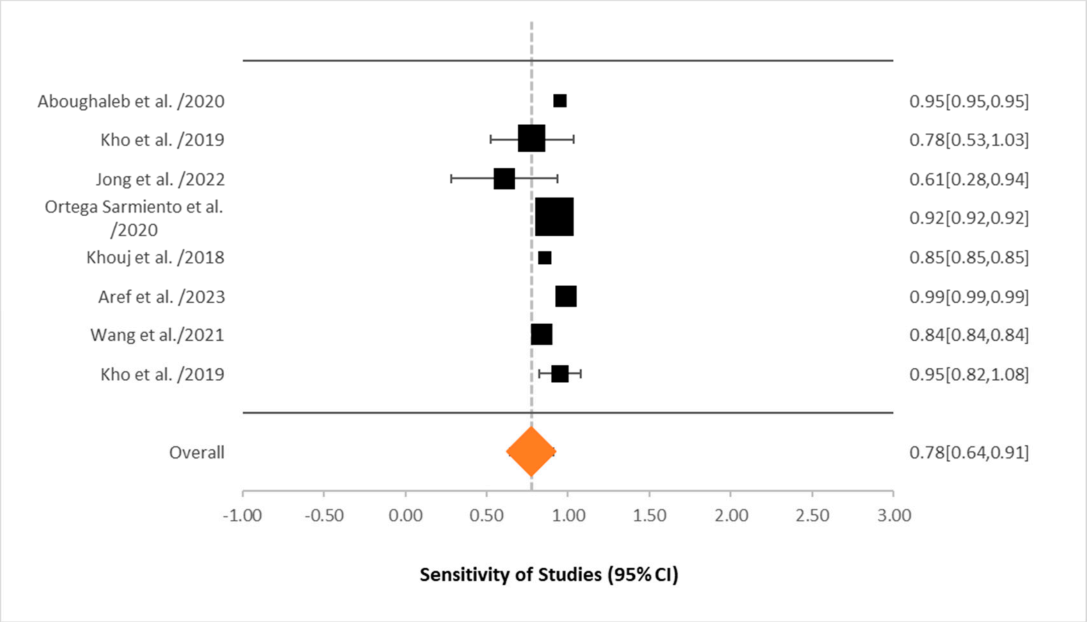

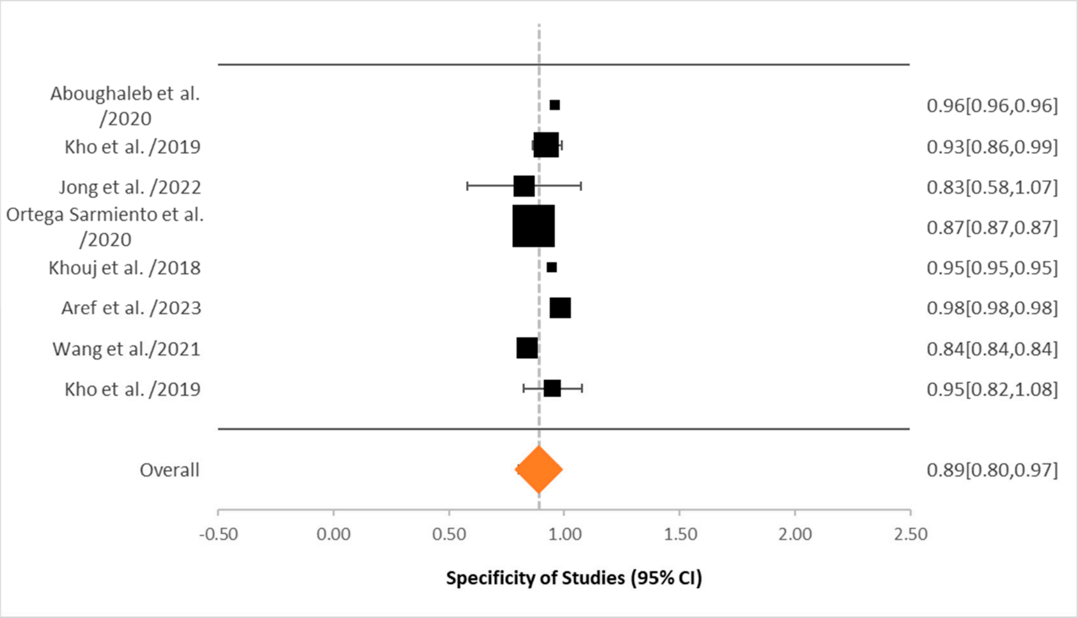

Figure S4. Sensitivity and Specificity Forest Plot (Studies)

S4. Deeks' Funnel Plot

This section presents Deeks' funnel plot based on different classifications such as nationality type, methods type, wavelength bands type, and published years. This funnel plot is capable of evaluating publication bias. It makes use of the dataset's square root and the diagnostic odds ratio. The regression line is also provided to ensure that the meta-analytical estimate of publication biases is taken into consideration.

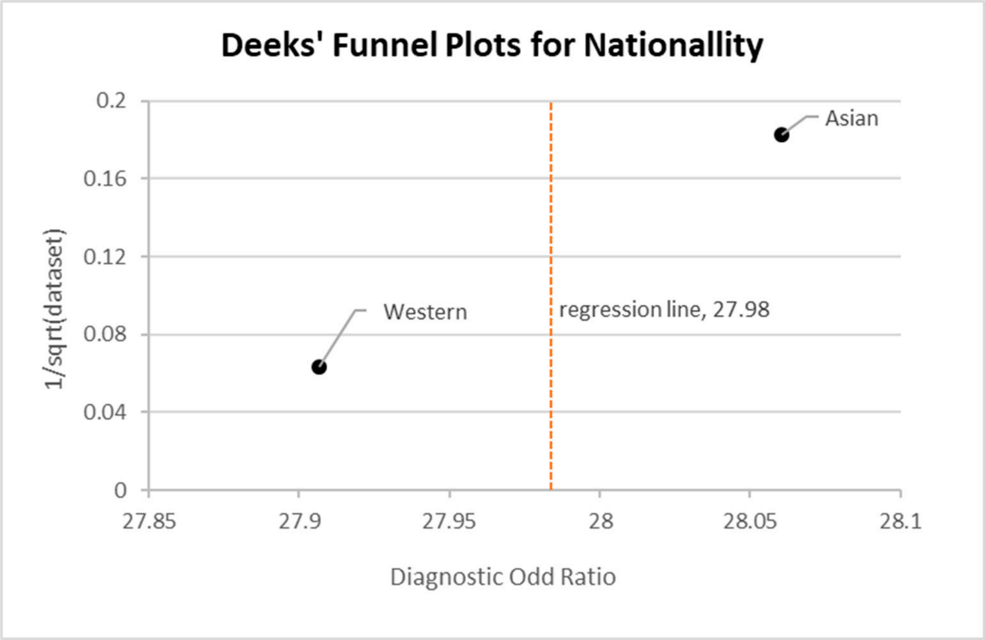

Figure S5. Deeks' Funnel Plot for Nationality

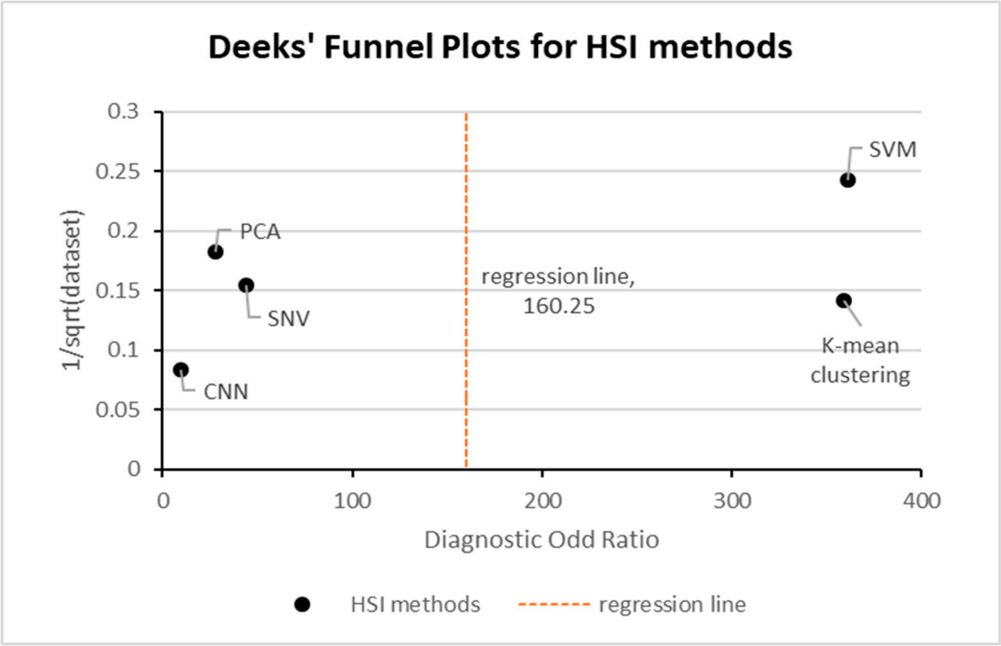

Figure S6. Deeks' Funnel Plot for HSI Methods

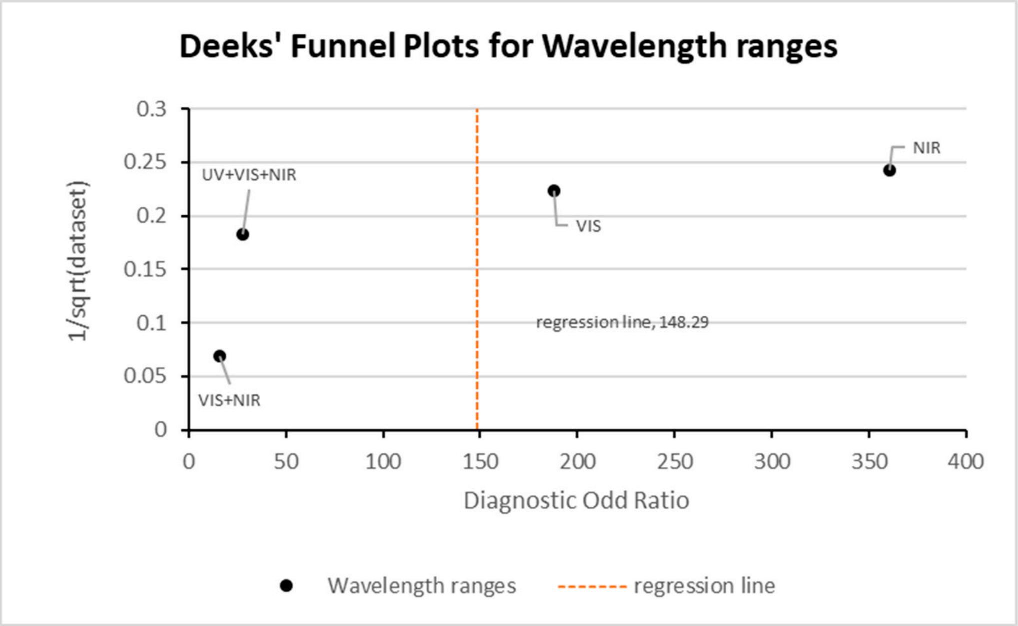

Figure S7. Deeks' Funnel Plot for Wavelength ranges

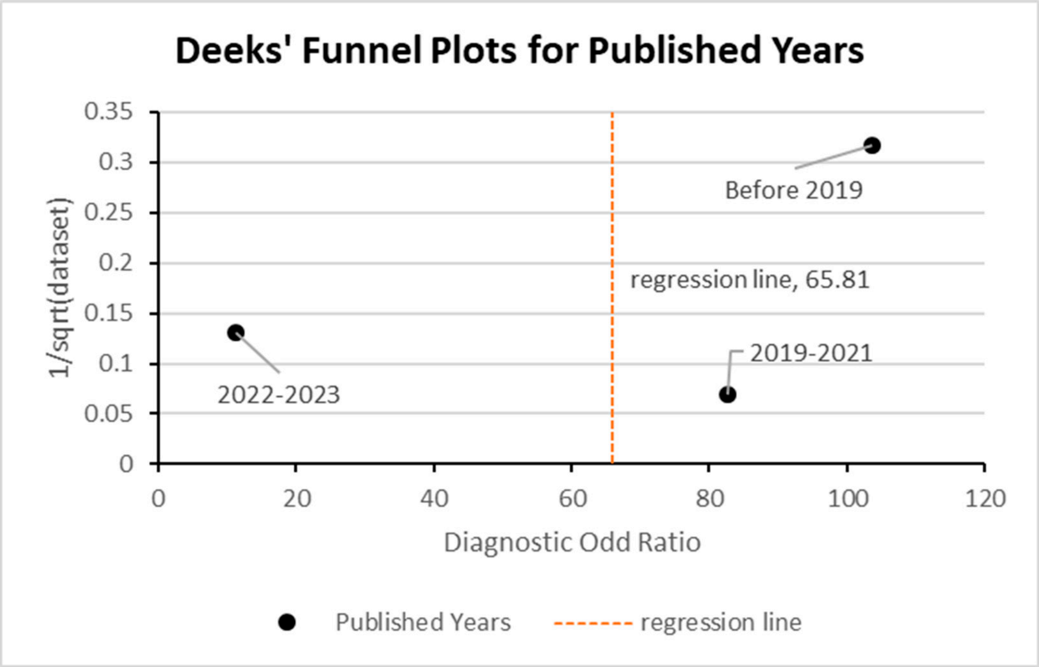

Figure S8. Deeks' Funnel Plot for Published years

S5. Summary of Computations for Forest Plots

This section shows the computations obtained for each forest plot. It contains the mean and confidence level that are essential in plotting the forest plots.

Sensitivity (K-mean clustering)		*Specificity (K-mean clustering)*	
Mean	0.960266667	Mean	0.934666667
Standard Error	0.012111335	Standard Error	0.040977975
Median	0.95	Median	0.96
Standard Deviation	0.020977448	Standard Deviation	0.070975935

Sample Variance	0.000440053	Sample Variance	0.005037583
Skewness	1.674840254	Skewness	-1.401554402
Range	0.038	Range	0.135
Minimum	0.9464	Minimum	0.8545
Maximum	0.9844	Maximum	0.9895
Sum	2.8808	Sum	2.804
Count	3	Count	3
Confidence Level(95.0%)	0.05211087	Confidence Level(95.0%)	0.176313996
**Lower**	0.908155796	**Lower**	0.75835267
**Upper**	1.012377537	**Upper**	1.110980663

Sensitivity (CNN)		*Specificity (CNN)*	
Mean	0.654285714	Mean	0.832857143
Standard Error	0.116166079	Standard Error	0.080702696
Median	0.72	Median	0.9
Standard Deviation	0.307346555	Standard Deviation	0.213519264
Sample Variance	0.094461905	Sample Variance	0.045590476
Kurtosis	4.572423377	Kurtosis	5.981074291
Skewness	-2.00168986	Skewness	-2.395951419
Range	0.92	Range	0.61
Minimum	0	Minimum	0.36
Maximum	0.92	Maximum	0.97
Sum	4.58	Sum	5.83
Count	7	Count	7
Confidence Level(95.0%)	0.284248154	Confidence Level(95.0%)	0.197472384
**Lower**	0.37003756	**Lower**	0.635384759
**Upper**	0.938533869	**Upper**	1.030329527

Sensitivity (SNV)		*Specificity (SNV)*	
Mean	0.78	Mean	0.925
Standard Error	0.02	Standard Error	0.005
Median	0.78	Median	0.925
Standard Deviation	0.028284271	Standard Deviation	0.007071068
Sample Variance	0.0008	Sample Variance	5E-05
Range	0.04	Range	0.01
Minimum	0.76	Minimum	0.92
Maximum	0.8	Maximum	0.93
Sum	1.56	Sum	1.85
Count	2	Count	2
Confidence Level(95.0%)	0.254124095	Confidence Level(95.0%)	0.063531024
**Lower**	0.525875905	**Lower**	0.861468976

**Upper**	1.034124095

**Upper**	0.988531024

Sensitivity (PCA)	
Mean	0.8412
Standard Error	0
Median	0.8412
Range	0
Minimum	0.8412
Maximum	0.8412
Sum	0.8412
Count	1
Confidence Level(95.0%)	#NUM!
**Lower**	0.8412
**Upper**	0.8412

Specificity (PCA)	
Mean	0.8412
Standard Error	0
Median	0.8412
Range	0
Minimum	0.8412
Maximum	0.8412
Sum	0.8412
Count	1
Confidence Level(95.0%)	#NUM!
**Lower**	0.8412
**Upper**	0.8412

Sensitivity (SVM)	
Mean	0.95
Standard Error	0.01
Median	0.95
Standard Deviation	0.014142136
Sample Variance	0.0002
Range	0.02
Minimum	0.94
Maximum	0.96
Sum	1.9
Count	2
Confidence Level(95.0%)	0.127062047
**Lower**	0.822937953
**Upper**	1.077062047

Specificity (SVM)	
Mean	0.95
Standard Error	0.01
Median	0.95
Standard Deviation	0.014142136
Sample Variance	0.0002
Range	0.02
Minimum	0.94
Maximum	0.96
Sum	1.9
Count	2
Confidence Level(95.0%)	0.127062047
**Lower**	0.822937953
**Upper**	1.077062047

Table S1. Sensitivity and Specificity Commutations for Forest Plot (HSI Method)

sensitivity Aboughaleb et al. /2020	
Mean	0.95
Standard Error	0
Median	0.95
Range	0
Minimum	0.95
Maximum	0.95
Sum	0.95
Count	1

specificity Aboughaleb et al. /2020	
Mean	0.96
Standard Error	0
Median	0.96
Range	0
Minimum	0.96
Maximum	0.96
Sum	0.96
Count	1

Confidence Level(95.0%)	#NUM!

Confidence Level(95.0%)	#NUM!

sensitivity Kho et al. /2019	
Mean	0.865
Standard Error	0.049916597
Median	0.87
Standard Deviation	0.099833194
Sample Variance	0.009966667
Kurtosis	-5.02719209
Skewness	-0.102512133
Range	0.2
Minimum	0.76
Maximum	0.96
Sum	3.46
Count	4
Confidence Level(95.0%)	0.15885689

specificity Kho et al. /2019	
Mean	0.9375
Standard Error	0.008539126
Median	0.935
Standard Deviation	0.017078251
Sample Variance	0.000291667
Kurtosis	0.342857143
Skewness	0.752837199
Range	0.04
Minimum	0.92
Maximum	0.96
Sum	3.75
Count	4
Confidence Level(95.0%)	0.027175309

sensitivity Jong et al. /2022	
Mean	0.654285714
Standard Error	0.116166079
Median	0.72
Standard Deviation	0.307346555
Sample Variance	0.094461905
Kurtosis	4.572423377
Skewness	-2.001689858
Range	0.92
Minimum	0
Maximum	0.92
Sum	4.58
Count	7
Confidence Level(95.0%)	0.284248154

specificity Jong et al. /2022	
Mean	0.832857143
Standard Error	0.080702696
Median	0.9
Standard Deviation	0.213519264
Sample Variance	0.045590476
Kurtosis	5.981074291
Skewness	-2.395951419
Range	0.61
Minimum	0.36
Maximum	0.97
Sum	5.83
Count	7
Confidence Level(95.0%)	0.197472384

sensitivity Ortega Sarmiento et al. /2020	
Mean	0.92
Standard Error	0
Median	0.92
Range	0
Minimum	0.92
Maximum	0.92
Sum	0.92

specificity Ortega Sarmiento et al. /2020	
Mean	0.87
Standard Error	0
Median	0.87
Range	0
Minimum	0.87
Maximum	0.87
Sum	0.87

Count	1		Count	1
Confidence Level(95.0%)	#NUM!		Confidence Level(95.0%)	#NUM!

sensitivity Khouj et al. /2018			*specificity Khouj et al. /2018*	
Mean	0.8545		Mean	0.9464
Standard Error	0		Standard Error	0
Median	0.8545		Median	0.9464
Range	0		Range	0
Minimum	0.8545		Minimum	0.9464
Maximum	0.8545		Maximum	0.9464
Sum	0.8545		Sum	0.9464
Count	1		Count	1
Confidence Level(95.0%)	#NUM!		Confidence Level(95.0%)	#NUM!

sensitivity Aref et al. /2023			*specificity Aref et al. /2023*	
Mean	0.9895		Mean	0.9844
Standard Error	0		Standard Error	0
Median	0.9895		Median	0.9844
Range	0		Range	0
Minimum	0.9895		Minimum	0.9844
Maximum	0.9895		Maximum	0.9844
Sum	0.9895		Sum	0.9844
Count	1		Count	1
Confidence Level(95.0%)	#NUM!		Confidence Level(95.0%)	#NUM!

sensitivity Wang et al./2021			*specificity Wang et al./2021*	
Mean	0.8412		Mean	0.8412
Standard Error	0		Standard Error	0
Median	0.8412		Median	0.8412
Range	0		Range	0
Minimum	0.8412		Minimum	0.8412
Maximum	0.8412		Maximum	0.8412
Sum	0.8412		Sum	0.8412
Count	1		Count	1
Confidence Level(95.0%)	#NUM!		Confidence Level(95.0%)	#NUM!

sensitivity Aboughaleb et al. /2020			*specificity Aboughaleb et al. /2020*	
Mean	0.95		Mean	0.96
Standard Error	0		Standard Error	0
Median	0.95		Median	0.96

Range	0	Range	0
Minimum	0.95	Minimum	0.96
Maximum	0.95	Maximum	0.96
Sum	0.95	Sum	0.96
Count	1	Count	1
Confidence Level(95.0%)	#NUM!	Confidence Level(95.0%)	#NUM!

sensitivity Kho et al. /2019		*specificity Kho et al. /2019*	
Mean	0.865	Mean	0.9375
Standard Error	0.049916597	Standard Error	0.008539126
Median	0.87	Median	0.935
Standard Deviation	0.099833194	Standard Deviation	0.017078251
Sample Variance	0.009966667	Sample Variance	0.000291667
Kurtosis	-5.02719209	Kurtosis	0.342857143
Skewness	-0.102512133	Skewness	0.752837199
Range	0.2	Range	0.04
Minimum	0.76	Minimum	0.92
Maximum	0.96	Maximum	0.96
Sum	3.46	Sum	3.75
Count	4	Count	4
Confidence Level(95.0%)	0.15885689	Confidence Level(95.0%)	0.027175309

sensitivity Jong et al. /2022		*specificity Jong et al. /2022*	
Mean	0.654285714	Mean	0.832857143
Standard Error	0.116166079	Standard Error	0.080702696
Median	0.72	Median	0.9
Standard Deviation	0.307346555	Standard Deviation	0.213519264
Sample Variance	0.094461905	Sample Variance	0.045590476
Kurtosis	4.572423377	Kurtosis	5.981074291
Skewness	-2.001689858	Skewness	-2.395951419
Range	0.92	Range	0.61
Minimum	0	Minimum	0.36
Maximum	0.92	Maximum	0.97
Sum	4.58	Sum	5.83
Count	7	Count	7
Confidence Level(95.0%)	0.284248154	Confidence Level(95.0%)	0.197472384

sensitivity Ortega Sarmiento et al. /2020		*specificity Ortega Sarmiento et al. /2020*	
Mean	0.92	Mean	0.87
Standard Error	0	Standard Error	0

Median	0.92
Range	0
Minimum	0.92
Maximum	0.92
Sum	0.92
Count	1
Confidence Level(95.0%)	#NUM!

sensitivity Khouj et al. /2018	
Mean	0.8545
Standard Error	0
Median	0.8545
Range	0
Minimum	0.8545
Maximum	0.8545
Sum	0.8545
Count	1
Confidence Level(95.0%)	#NUM!

sensitivity Aref et al. /2023	
Mean	0.9895
Standard Error	0
Median	0.9895
Range	0
Minimum	0.9895
Maximum	0.9895
Sum	0.9895
Count	1
Confidence Level(95.0%)	#NUM!

sensitivity Wang et al./2021	
Mean	0.8412
Standard Error	0
Median	0.8412
Range	0
Minimum	0.8412
Maximum	0.8412
Sum	0.8412
Count	1
Confidence Level(95.0%)	#NUM!

Median	0.87
Range	0
Minimum	0.87
Maximum	0.87
Sum	0.87
Count	1
Confidence Level(95.0%)	#NUM!

specificity Khouj et al. /2018	
Mean	0.9464
Standard Error	0
Median	0.9464
Range	0
Minimum	0.9464
Maximum	0.9464
Sum	0.9464
Count	1
Confidence Level(95.0%)	#NUM!

specificity Aref et al. /2023	
Mean	0.9844
Standard Error	0
Median	0.9844
Range	0
Minimum	0.9844
Maximum	0.9844
Sum	0.9844
Count	1
Confidence Level(95.0%)	#NUM!

specificity Wang et al./2021	
Mean	0.8412
Standard Error	0
Median	0.8412
Range	0
Minimum	0.8412
Maximum	0.8412
Sum	0.8412
Count	1
Confidence Level(95.0%)	#NUM!

Table S2. Sensitivity and Specificity Computations for Forest Plot (Studies)

Sensitivity Nationality	
Mean	0.807528571
Standard Error	0.033671429
Median	0.807528571
Standard Deviation	0.047618591
Sample Variance	0.00226753
Range	0.067342857
Minimum	0.773857143
Maximum	0.8412
Sum	1.615057143
Count	2
Confidence Level(95.0%)	0.427836065

Specificity Nationality	
Mean	0.865985714
Standard Error	0.024785714
Median	0.865985714
Standard Deviation	0.035052293
Sample Variance	0.001228663
Range	0.049571429
Minimum	0.8412
Maximum	0.890771429
Sum	1.731971429
Count	2
Confidence Level(95.0%)	0.31493236

Sensitivity METHODS	
Mean	0.83136381
Standard Error	0.053925919
Median	0.8412
Standard Deviation	0.120582021
Sample Variance	0.014540024
Kurtosis	-0.383482752
Skewness	-0.717303953
Range	0.295714286
Minimum	0.654285714
Maximum	0.95
Sum	4.156819048
Count	5
Confidence Level(95.0%)	0.149722354

Specificity METHODS	
Mean	0.902531429
Standard Error	0.027480468
Median	0.925
Standard Deviation	0.061448195
Sample Variance	0.003775881
Kurtosis	-3.006434172
Skewness	-0.400261012
Range	0.130742857
Minimum	0.832857143
Maximum	0.9636
Sum	4.512657143
Count	5
Confidence Level(95.0%)	0.076298011

Sensitivity BANDS	
Mean	0.8516
Standard Error	0.051299744
Median	0.871725
Standard Deviation	0.102599488
Sample Variance	0.010526655
Kurtosis	0.635259661
Skewness	-0.977630964
Range	0.23705

Specificity BANDS	
Mean	0.90271
Standard Error	0.028700431
Median	0.90822
Standard Deviation	0.057400863
Sample Variance	0.003294859
Kurtosis	-5.04233861
Skewness	-0.162033926
Range	0.112

Minimum	0.71295		Minimum	0.8412
Maximum	0.95		Maximum	0.9532
Sum	3.4064		Sum	3.61084
Count	4		Count	4
Confidence Level(95.0%)	0.163258681		Confidence Level(95.0%)	0.091337582

Sensitivity YEARS			*Specificity YEARS*	
Mean	0.800104762		Mean	0.904304762
Standard Error	0.068394124		Standard Error	0.028803332
Median	0.8545		Median	0.917314286
Standard Deviation	0.118462098		Standard Deviation	0.049888834
Sample Variance	0.014033269		Sample Variance	0.002488896
Skewness	-1.630633614		Skewness	-1.093669113
Range	0.217385714		Range	0.0972
Minimum	0.664214286		Minimum	0.8492
Maximum	0.8816		Maximum	0.9464
Sum	2.400314286		Sum	2.712914286
Count	3		Count	3
Confidence Level(95.0%)	0.294276165		Confidence Level(95.0%)	0.123930735

Sensitivity OVERALL			*Specificity OVERALL*	
Mean	0.778346667		Mean	0.887466667
Standard Error	0.062421186		Standard Error	0.039374726
Median	0.8412		Median	0.94
Standard Deviation	0.241756214		Standard Deviation	0.152497657
Sample Variance	0.058446067		Sample Variance	0.023255535
Kurtosis	8.22851277		Kurtosis	12.00936986
Skewness	-2.613064507		Skewness	-3.343609313
Range	0.9895		Range	0.6244
Minimum	0		Minimum	0.36
Maximum	0.9895		Maximum	0.9844
Sum	11.6752		Sum	13.312
Count	15		Count	15
Confidence Level(95.0%)	0.133880129		Confidence Level(95.0%)	0.084450387

Table S3. Sensitivity and Specificity Computations for Meta Regression

S6. Summary of Computations for Deeks' Funnel Plots

This section shows the computations obtained for each Deeks' funnel plot. It contains the regression statistics and the number of observations needed in the funnel plot.

SUMMARY OUTPUT (studies)

Regression Statistics

Multiple R	0.089092
R Square	0.007937
Adjusted R Square	-0.15741
Standard Error	0.083495
Observations	8

Table S4. Regression Statistics (Studies)

SUMMARY OUTPUT (Nationality)

Regression Statistics	
Multiple R	1
R Square	1
Adjusted R Square	65535
Standard Error	0
Observations	2

Table S5. Regression Statistics (Nationality)

SUMMARY OUTPUT (HSI methods)

Regression Statistics	
Multiple R	0.518839258
R Square	0.269194176
Adjusted R Square	0.025592234
Standard Error	0.057213811
Observations	5

Table S6. Regression Statistics (HSI Methods)

SUMMARY OUTPUT (Wavelength ranges)

Regression Statistics	
Multiple R	0.777193084
R Square	0.60402909
Adjusted R Square	0.406043635
Standard Error	0.060101977
Observations	4

SUMMARY OUTPUT (Published year)

Regression Statistics	
Multiple R	0.481461
R Square	0.231804
Adjusted R Square	-0.53639
Standard Error	0.159678
Observations	3

Table S7. Regression Statistics (Wavelength ranges)

S7. Deeks' Funnel Plot Computation of p-value

This section shows the computations obtained for the p value needed in the Deeks' funnel plot. A p-value is essential in determining the heterogeneity of the data involved.

P-values below 0.05 suggest heterogeneity. By contrast, p-values above 0.05 suggest no heterogeneity.

ANOVA

Source of Variation	*SS*	*df*	*MS*	*F*	*P-value*	*F crit*
Between Groups	0.101126	7	0.014447	2.924354	0.077901	3.500464
Within Groups	0.039521	8	0.00494			
Total	0.140646	15				

Table S8. P-value of Deeks' Funnel Plot (Studies)

ANOVA

Source of Variation	*SS*	*df*	*MS*	*F*	*P-value*	*F crit*
Between Groups	7.9E-05	1	7.9E-05	0.023105189	0.89313253	18.51282
Within Groups	0.006834	2	0.003417			
Total	0.006913	3				

Table S9. P-value of Deeks' Funnel Plot (Nationality)

ANOVA

	df	*SS*	*MS*	*F*	*Significance F*	*P-value*
Regression	1	0.003617	0.003617	1.105058	0.370362	0.03294
Residual	3	0.00982	0.003273			
Total	4	0.013438				

Table S10. P-value of Deeks' Funnel Plot (HSI Methods)

ANOVA

	df	*SS*	*MS*	*F*	*Significance F*	*P-value*
Regression	1	0.01102052	0.01102052	3.050876088	0.222806916	0.105336895
Residual	2	0.007224495	0.003612248			
Total	3	0.018245015				

Table S11. P-value of Deeks' Funnel Plot (Wavelength ranges)

ANOVA

Source of Variation	*SS*	*df*	*MS*	*F*	*P-value*	*F crit*
Between Groups	0.02736037	2	0.013680186	1.868	0.297221601	9.552094
Within Groups	0.02197042	3	0.007323472			
Total	0.04933079	5				

Table S12. P-value of Deeks' Funnel Plot (Published Years)

ANOVA

Source of Variation	*SS*	*df*	*MS*	*F*	*P-value*	*F crit*
Between Groups	0.001863069	3	0.000621	0.226321	0.873839312	6.59138212
Within Groups	0.01097597	4	0.002744			
Total	0.012839039	7				

Table S13. P-value of Deeks' Funnel Plot (Classification)
